# Bamboo Hair in Netherton's Syndrome

**DOI:** 10.4103/0974-7753.58561

**Published:** 2009

**Authors:** Atul D Salodkar, Sanjiv V Choudhary, Gori Jadwani, Adarshlata Singh

**Affiliations:** Department of Dermatology, Jawaharlal Nehru Medical College, Sawangi, Wardha [M.S], India

Sir,

Netherton's syndrome is a rare autosomal recessive genodermatosis of unknown cause characterized by erythroderma, trichorrhexis invaginata (bamboo hair), ichthyosis linearis circumflexa and atopic diathesis. In 1949, Comel[1] first coined the term ichthyosis linearis circumflexa. In 1958, Netherton[2] described a young girl with generalized scaly dermatitis and fragile nodular hair-shaft deformities that he termed trichorrhexis nodosa. Later, this was more appropriately renamed as trichorrhexis invaginata (bamboo hair). In 1974, Mevorah *et al*.[3] established the clinical relationship between ichthyosis linearis circumflexa and Netherton's syndrome. The atopic diathesis occurs in approximately 75% of patients with Netherton's syndrome. This syndrome is caused by mutation in the Spinks gene, which is located on the long arm of Chromosome 5.[4] This gene codes for production of a protein LEKTI, which inhibits the enzyme serine proteinase in the outermost layer of the skin. The function of this enzyme is to break down the intracellular cement leading to desquamation of epidermal cells. A LEKTI deficiency leads to an uninhibited desquamation of horny cells; as a result, the skin becomes red and scaly. This is responsible for all the characteristic symptoms of Netherton's syndrome.

**Figure 1:**
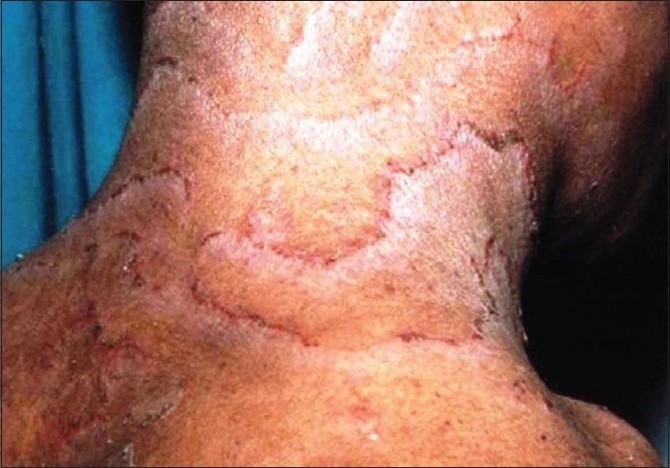
Erythematous annular and polycyclic, scaly patches with double edged scales at the periphery of the lesions involving neck

**Figure 2:**
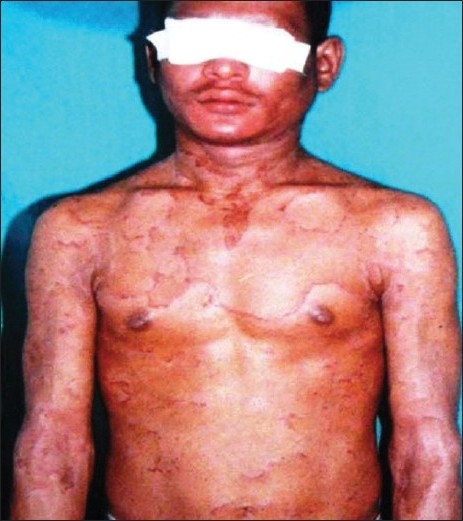
Annular and polycyclic, scaly patches with double edged scales at the periphery of the lesions involving trunk and extremities

**Figure 3:**
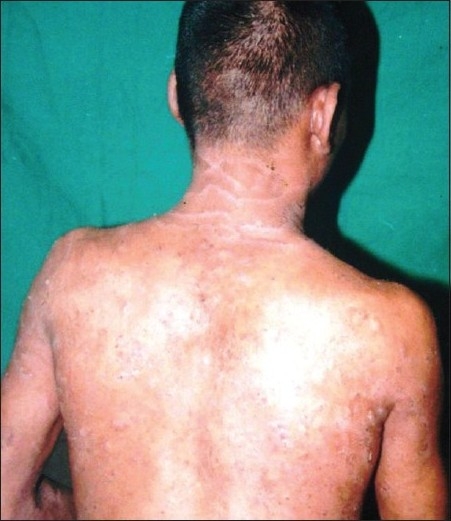
Annular and polycyclic, scaly patches with double edged scales at the periphery of the lesions involving back

**Figure 4:**
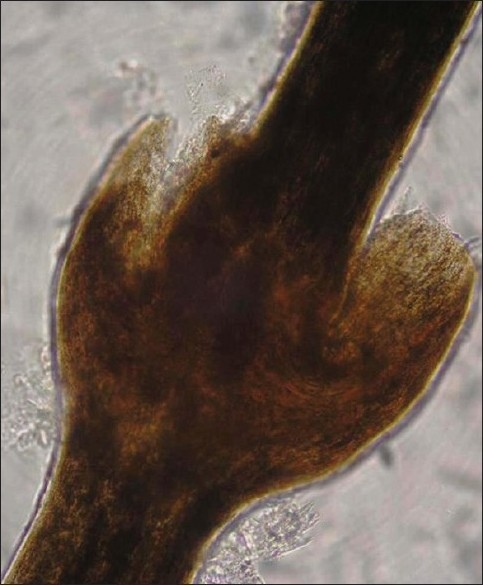
The light microscopic examination of scalp hair showed the typical trichorrhexis invaginata (bamboo hair)

A 24-year-old male patient presented with generalized scaly lesions since birth. History of frequent exacerbations and complete remissions of skin lesions was present since childhood. Personal history of atopy in the form of recurrent allergic rhinitis was present since childhood. History of parental consanguinity was not present. There was no history of fluid-filled lesions. On general examination, patient was moderately built and nourished. Cutaneous examination revealed multiple widespread erythematous annular and polycyclic, scaly patches with double-edged scales at the periphery of the lesions [Figures 1‐3], involving the trunk and extremities. Skin lesions were continuously changing their shape and size during each exacerbations and leaving no scarring or pigmentary changes upon healing. Nails were shiny. Scalp hairs were sparse, rough and lusterless. Mucus membrane and other systemic examination were within normal limits. Blood investigations revealed hemoglobin-9.5 gm%, white blood count-7300 cells/ m^3^, mild eosinophilia, raised erythrocyte sedimentation rate-54 mm/h and raised serum IgE level-11412.30 IU/ ml. The light microscopic examination of scalp hairs showed the typical trichorrhexis invaginata (bamboo hair), with the distal portion of the shaft invaginated into the proximal portion [Figure 4]. Biopsy taken from the lesion on the back was consistent with the clinical diagnosis of ichthyosis linearis circumflexa.

On the bases of ichthyosis linearis circumflexa, characteristic hair anomaly and atopic manifestations [recurrent allergic rhinitis and raised IgE level], a diagnosis of Netherton's syndrome was made.

Netherton's syndrome is characterized by the triad of ichthyosis linearis circumflexa, trichorrhexis invaginata (bamboo hair) and an atopic diathesis.[5] Our case had classical cutaneous lesions of ichthyosis linearis circumflexa in the form of recurrent crops of erythematous annular and polycyclic scaly patches with double-edged scales, which constantly change their size and shape and involutes spontaneously. Our case also had the trichorrhexis invaginata involving scalp hairs, which is pathognomonic of Netherton's syndrome and its presence confirms the diagnosis. Personal or family history of atopy is another association.[6] From 30 to 75% of patients with Netherton's syndrome develop atopic manifestations such as atopic dermatitis-like skin lesions, urticaria, angioneurotic edema, asthma, allergic rhinitis, food allergy, peripheral eosinophilia and elevated serum IgE level. Other associated manifestations of Netherton's syndrome include aminoaciduria, failure to thrive, mental and neurological retardation and immune abnormalities[3,7,8] In our patient, atopic manifestations were present in the form of recurrent allergic rhinitis, peripheral eosinophilia and grossly elevated serum IgE level. Netherton's syndrome has been observed almost always in females. Smith *et al*. in his review of 43 patients described a male patient[8] In our case report also, all the manifestations of Netherton's syndrome was seen in a male patient. Teeth and nails are not affected in this syndrome. Though mental retardation has been reported in some children,[9] our patient had normal intelligence and had normal teeth and nails.

We report this case for its rarity and classical presentation.
